# A case of eccrine porocarcinoma characterized by a progressive increase in the level of Ki-67 index: case report and review of literature

**DOI:** 10.1186/s12893-019-0595-4

**Published:** 2019-10-10

**Authors:** Jie Shen, Xinfa Pan, Yongfu Lu, Desheng Pan, Yuehui Ma, Renya Zhan

**Affiliations:** 1 Department of Neurosurgery, The First Affiliated Hospital, College of Medicine, Zhejiang University, Zhejiang, 310003 Hangzhou China; 2 Department of Pathology, The First Affiliated Hospital, College of Medicine, Zhejiang University, Zhejiang, 310003 Hangzhou China

**Keywords:** Eccrine porocarcinoma, Hidradenocarcinoma, Sweat gland carcinoma, Scalp, Ki-67, Metastasis, Invasion, Dura

## Abstract

**Background:**

Eccrine porocarcinoma is an extremely rare skin adnexal malignant neoplasia with highly invasive and metastatic potential. We report an additional case of eccrine porocarcinoma with intracranial metastases. This case is characterized by a complete record of the progress of eccrine porocarcinoma, its immunohistochemistry after three operations showed a progressive increase in the level of Ki-67 index.

**Case presentation:**

We herein report a case of a 37-year-old-male with eccrine carcinoma occurring on the left posterior occipital scalp which invaded the skull and dura, presenting with progressive headache. This patient has performed three surgeries in total. During the last hospitalization, he underwent an extended surgical resection, lymphadenectomy, myocutaneous flap transplantation and vascular anastomosis in our institution. After surgery, he was treating with radiotherapy at 200 Gray in 12 fractions. But one year after the operation, he developed chest tightness, imaging examination and biopsy puncture revealed pulmonary metastasis.

**Conclusion:**

Intracranial metastasis of eccrine porocarcinoma is a late event with poor prognosis. This case emphases on that progressively increased level of Ki-67 index may predict more chance to occur the intracranial metastasis of scalp eccrine porocarcinoma, long-term follow-up and appropriately dense follow-up interval is necessary.

## Background

Eccrine porocarcinoma or malignant eccrine carcinoma, is a rare malignant tumor of the skin adnexa with highly invasive and metastatic potential, occurring mainly in the dense regions of eccrine sweat gland, accounting for about 0.005% of all malignant epithelial tumors, presenting approximate morbidity in genders [1–3]. The specific etiology of eccrine porocarcinoma is not yet clear, local skin irritation, X-ray irradiation may be its cause. Local repeated recurrence and tumor metastasis are major clinical features of eccrine porocarcinoma [4], tumor tissue usually metastasize to local lymph nodes or adjacent cutaneous, and less common distant organs for metastasis include the breast, liver, lungs, retroperitoneum, ovaries [5]. In general, the recurrence rate and lymph node metastasis rate of eccrine porocarcinoma patients were 20% respectively [6, 7]. At present, the treatment of sweat gland carcinoma (SGC) is still constantly evolving, but its first choice is to take a surgical resection [8]. However, there is no final conclusion about whether radiotherapy or chemotherapy is efficient for sweat gland carcinoma. As review of the literatures revealed, there are only a total of 5 cases about intracranial metastasis and invasion of scalp sweat gland carcinoma were reported to date (Table 1) [3, 9, 10]. We report an additional case of eccrine porocarcinoma with intracranial metastases. Besides, review some existing literatures about this disease and its management.

**Table 1:** Overview of published cases of sweat gland carcinoma with intracranial metastases

Reference	Gender	Age	Primary site	Treatment	Intracranial metastasis	Follow-up
Kim et al. [9]	Male	42	right palm.	surgical resection lymphadenectomy knife radiosurgery radiotherapy	Yes	not informed
Omer Waqas et al. [10] Case 1	Female	43	occipital scalp	surgical resection lymphadenectomy radiotherapy	Yes	6 months
Case 2	Male	75	not informed	radiotherapy	Yes	2 years
Case 3	Male	61	Left temporal region	surgical resection radiotherapy	Yes	not informed
Misbahuddin et al. [3]	Male	67	Parietal scalp	surgical resection lymphadenectomy Radiotherapy chemotherapy	Yes	4 years

## Case presentation

**Fig. 1:**
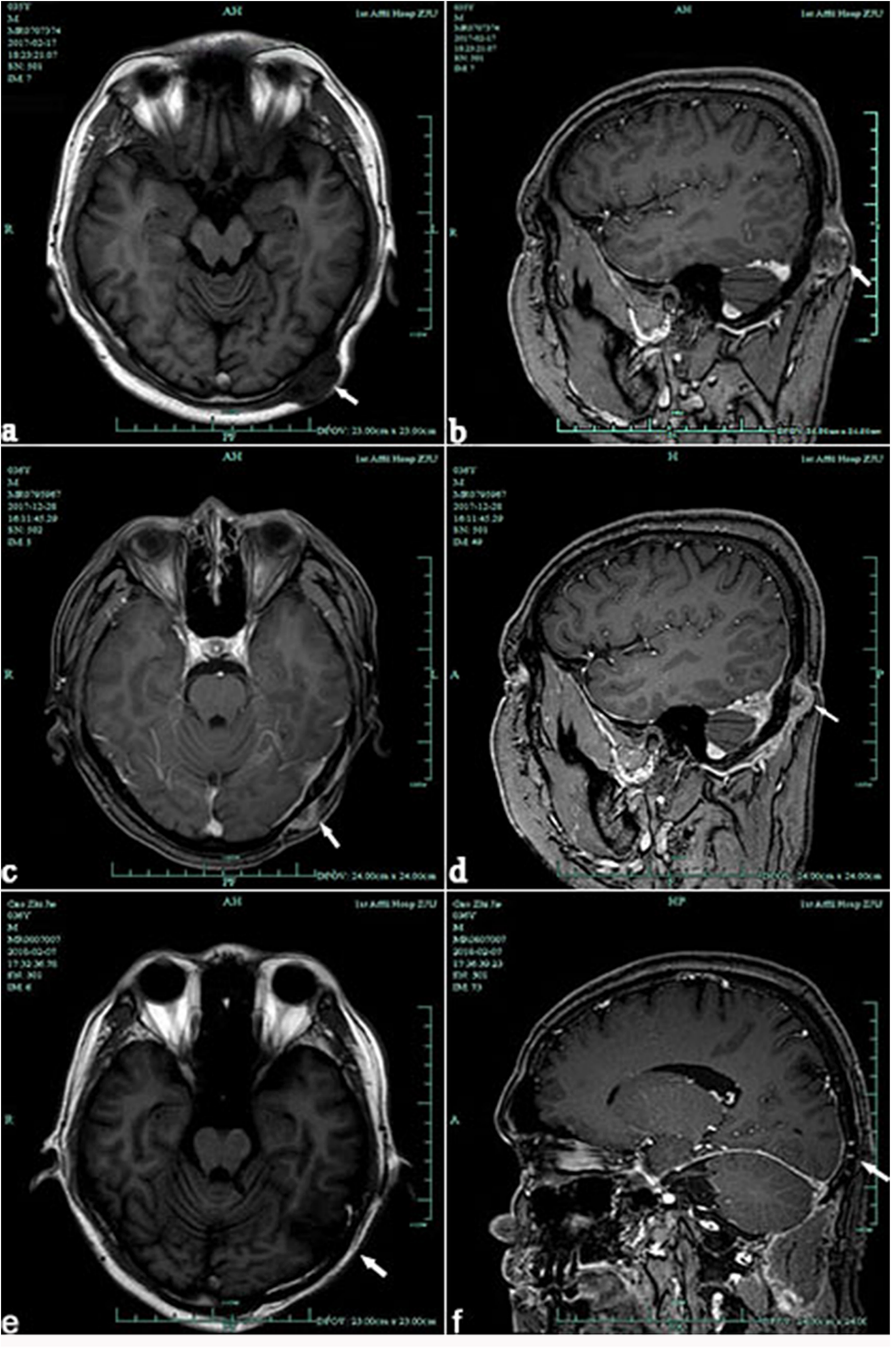
(**a**, **b**) A circular abnormal signal is seen on the left occipital scalp. The border of lesion is clear and its size is about 3.3 × 1.8 cm, the adjacent bone is thin. (**c**,**d**) There are enhanced abnormal signals on the left occipital skin, subcutaneous and adjacent meninges. (**e**,**f**) Postoperative examination of MRI image showing cranial screw fixation, no recurrence

A 37-year-old man developed a gradually growing nodule on the left posterior occipital scalp, which he had first noticed 2 years previously. Excision of scalp mass was performed in local hospital and the postoperative pathology showed skin attachment malignancy. In February 2017, this patient addressed to our hospital for the recurrence of lesion. Brain MRI (Fig. 1. a, b) suggested that soft tissue tumor of the left occipital superior scalp. Then we completed a wide local excision of the posterior occipital region of scalp and grind local skull that has been invaded by tumor. The pathological diagnosis of the mass was eccrine porocarcinoma. No postoperative radiotherapy or chemotherapy was given after two surgery operation.

**Fig. 2:**
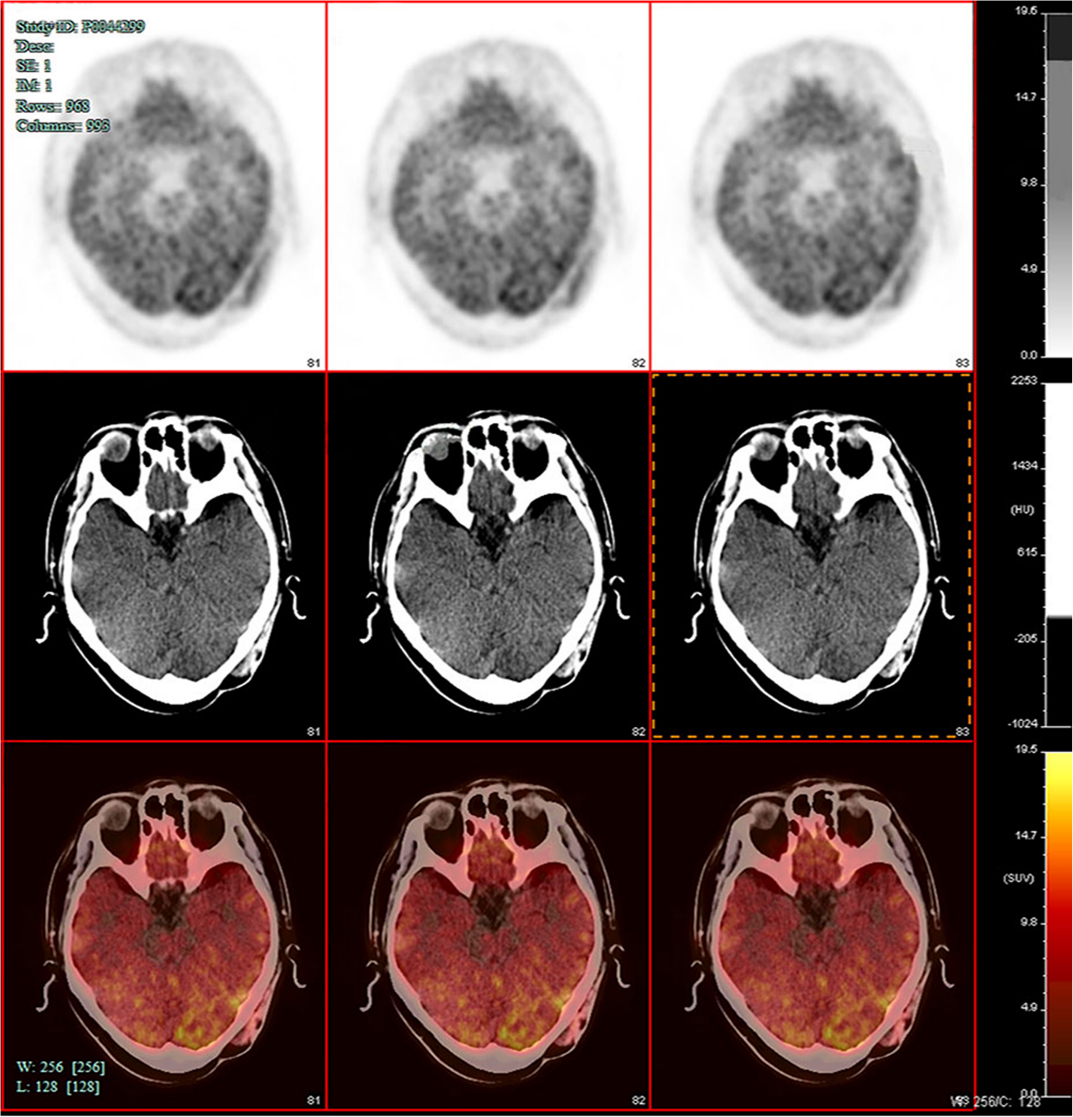
Left occipital poorly differentiated carcinoma after secondary resection: left occipital are scalp and subcutaneous soft tissue density irregularity, with left occipital bone destruction, FDG metabolism increased, consider tumor recurrence

**Fig. 3:**
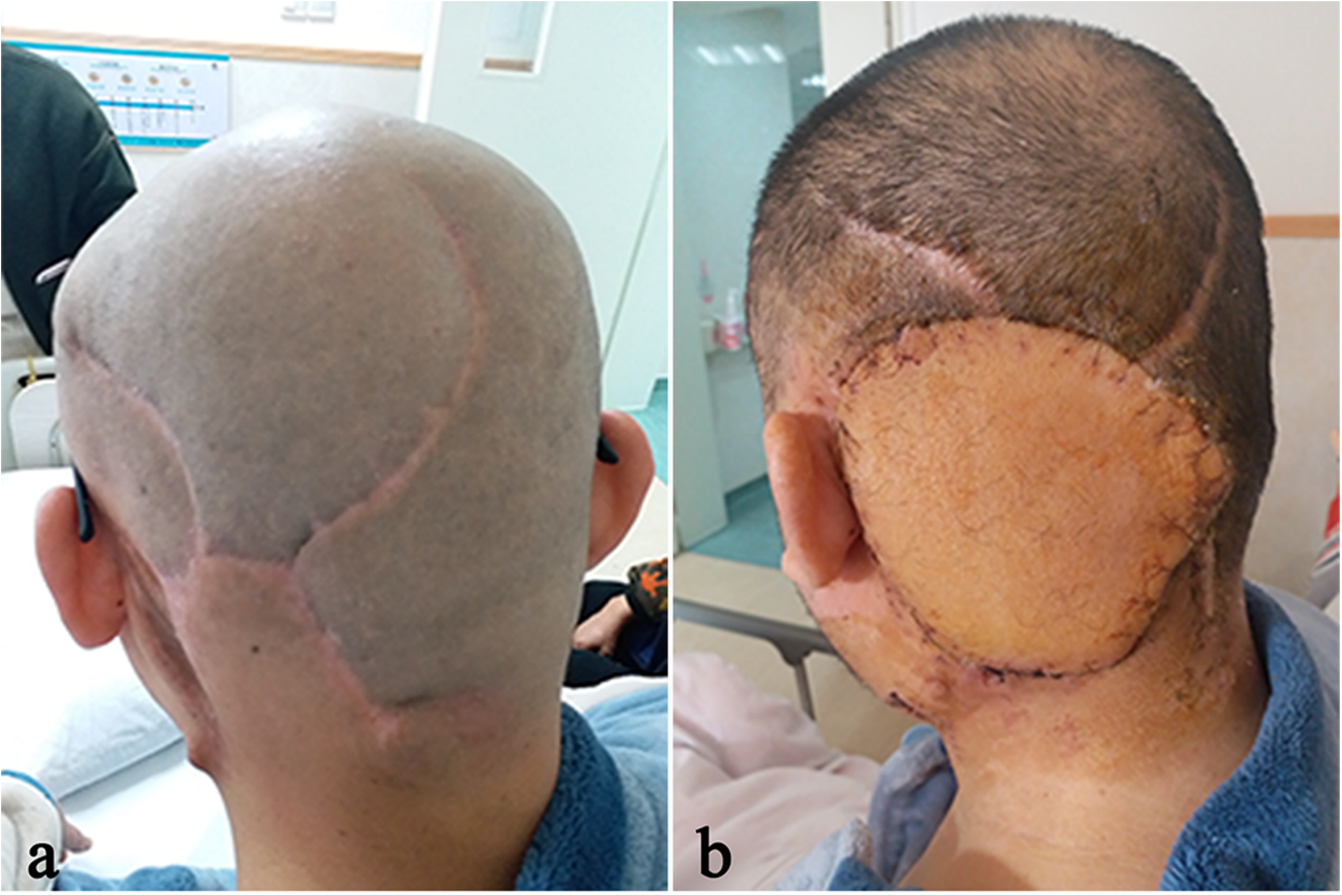
(**a**) image of the surgical area before the last operation. (**b**) the postoperative image of transferred skin flaps

**Fig. 4:**
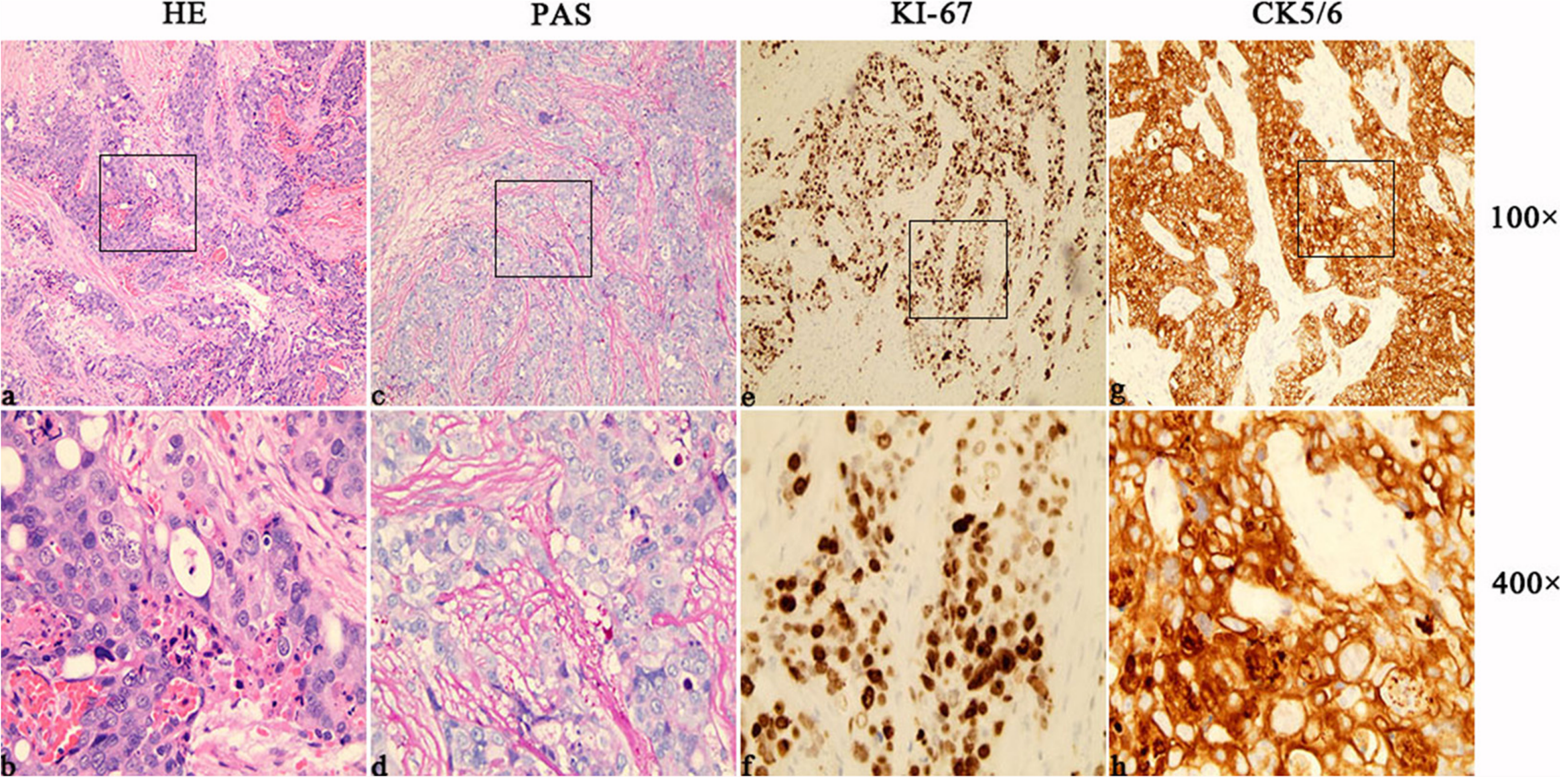
(**a**, **b**) Histopathology revealed cells contained atypical cell nuclei with conspicuous nucleoli, and small amount of eosinophilic cytoplasm. Mitotic figures and apoptotic cells were existed in the lesion (original magnification × 100 or 400, H&E). (**c**, **d**, **e**, **f**, **g**, **h**) Immunohistochemically, the lesional cells were positive when stained with antibodies against cytokeratin 5/6, Ki-67, and PAS, highlighted scattered ductal structures throughout the neoplasm

More than seven months after the operation in our hospital, patient felt pain on the left side of the top occipital area. Head MRI (Fig. 1. c, d) suggested that recurrence and intracranial metastasis of the tumor, and adjacent cranium is involved. PET/CT showed that left occipital area’s FDG metabolism increased, consider tumor recurrence (Fig. 2). Other area showed no abnormal increase in metabolism of FDG. After informed consent, extended surgical resection was taken. We excised the local skull and dura both invaded by tumor. Last, myocutaneous flap transplantation and revascularization was performed (Fig. 3). The pathologic diagnosis was eccrine porocarcinoma (Fig. 4). This patient accepted radiotherapy at 200 Gy in 12 fractions after the surgery and showed no signs of recurrence 6 months after the surgery (Fig. 1. e, f). The positive results of immunohistochemistry after three surgery operation were showed in Table 2. One year after the operation, this patient developed chest tightness, imaging examination and biopsy puncture revealed pulmonary metastasis. And he accepts palliative treatment nowadays.

**Table 2:** Pathological results and location of metastasis after three operations

Hospital	Time	Clinical symptoms	Pathology	Immunohistochemistry	Indicator of progressive increase	Tumor metastasis
Local hospital	2015	A painless mass	Sweat gland carcinoma	CK5/6(+), CK-20(−), CEA(−), BCL-2(+, 2%), KI-67(2 + , 60%), EMA(++)	KI-67(+, 60%)	No metastasis
First Affiliated Hospital of Medical College of Zhejiang University	2017	A painless mass	malignant eccrine carcinoma	CK5/6(+), CK7(+), CK20(+), P63(−), MMG(−), S-100(−), Ki-67(+, 70%),G15(−), CD43(−), PAS(+)	Ki-67(+, 70%)	Local skull metastasis
First Affiliated Hospital of Medical College of Zhejiang University	2018	Mass with tenderness Headache Intracranial hypertension	malignant eccrine carcinoma	CK5/6(+), CK7(+), CK20(−), P63(−), MMG(−), S-100(−), Ki-67(+, > 60%),G15(−), CD43(−), GCDFP15(−)	Ki-67(++, > 60%)	Local skull metastasis Dura metastasis

## Discussion and conclusions

Eccrine porocarcinoma is a rare clinical tumor, tends to arise in areas with high concentration of apocrine sweat glands, like the axilla, followed by lower extremities (35%), head and neck (24%) and upper extremities (14%) [2, 11]. Its clinical manifestation is mostly a painless slow-growing subcutaneous solitary mass, but sometimes, tumor may rapidly expand in a short term. Neoplasms usually are normal skin color, maybe light red or purple. And tumor tissue adhered to the skin surface, few cases presented local ulceration [12]. In 1969, Mishima and Marioka formally proposed the term eccrine porocarcinoma [13]. Eccrine porocarcinoma has a high rate of recurrence and metastatic, its mortality rate of metastatic cases increased significantly to 80% with distant metastasis and 65% with lymph nodes [11].

At present, the treatment of eccrine porocarcinoma still follows the principle of sweat gland carcinoma. Mohs micrographic surgery (MMS) or complete circumferential peripheral and deep margin assessment (CCPDMA) are recommended as first-line treatment for patient who is suspected to have sweat gland carcinoma or pathologically confirmed sweat gland carcinoma. Wide local excision can be performed in lieu of MMS and CCPDMA, but the extent of resection should be at least 2 cm from the edge [14]. The resection margin needs to be diagnosed pathologically, or it is necessary to send surgical margin tissue to frozen examination to improve the thoroughness of resection. Due to the fact that primary lesion cannot be directly sutured after resection, skin grafting and partial flap repairing should be given [8, 15].

Status of lymph nodes can be used as an important factor to observe the prognosis of SGC. Lymph node biopsy and resection has clear guiding significance for comprehensive treatment and prognosis of early SGC with regional lymph node metastasis [16, 17]. Another retrospective research performed an analysis of 186 patients: 96% of patients underwent surgical resection or extended resection, 69% of patients underwent lymph node dissection. The median survival time was approximately 51.5 months. Patient’s survival time was significantly shorter who with lymph node metastasis or distant metastasis [18].

The efficacy of radiotherapy and chemotherapy for SGC remains controversial [19]. Some studies pointed out radical radiotherapy could be given after surgery to reduce local recurrence and control distant metastasis of easy-relapsed tumors [20]. In another research, 2 cases of SGC patients with axillary lymph node positive received 50Gy/25f adjuvant radiotherapy after surgery. No recurrence after following up for 9 months and 10 months, respectively [21]. Recent studies found that some patients with hidradenocarcinoma showed positive expression of hormone receptor ER, PR, AR, and HER-2 [22]. Schroder et al. performed tamoxifen treatment in a 64-year-old woman with ER-positive hidradenocarcinoma and achieved complete relief for up to 3 years [23]. These researches suggest in hidradenocarcinoma with positive expression of ER or PR, tamoxifen and trastuzumab can be used as treatment options. And another case of patient with metastatic SGC suggested that the combination of pertuzumabbased targeted therapy with taxane chemotherapy might develop into a new treatment option [24]. In addition, topical Aminolevulinic Acid Photodynamic therapy could be an effective treatment method to improve surgical outcomes of hidradenocarcinoma [25].

In this case, recurrence time of the tumor were fourteen months and ten months, respectively. Because of the diffuse infiltrative growth of sweat gland carcinoma tissue, so surgical resection cannot effectively control the recurrence of sweat gland carcinoma [26]. And the absence of chemotherapy or radiotherapy in previous hospitalizations may connect with its recurrence. It is worth mentioning that immunohistochemistry after three operations showed a progressive increase in the level of Ki-67 index (Table 2). Ki-67 is usually overexpressed in malignant soft tissue tumors and is associating with tumor development, infiltration, metastasis, and prognosis [27]. The high level of Ki-67 index may predict its recurrence and metastasis.

In conclusion, the intracranial metastasis of eccrine porocarcinoma is a late event with a poor prognosis. Summarize experience from this case and previous literature, an extended surgical resection is necessary to improve prognosis. Lymphadenectomy is recommended to perform if there are palpable enlarged lymph nodes in the clinical or imaging evidence indicates local lymph node metastasis. And radiotherapy and chemotherapy can be used to compensate for the disadvantages of insufficient surgical resection. If patient is diagnosed as scalp sweat gland carcinoma with a high level of Ki-67 index, prolonging follow-up time and shortening the follow-up interval is mandatory.

## Consent

Written consent was obtained from the patient for publication of this case report and any accompanying images. A copy of the written consent is available for review by the Editor-in-Chief of this journal.

## Data Availability

All data of this patient of this case report is included in this published article.

## Abbreviations

BCL-2: B cell lymphoma-2
CCPDMA: Complete circumferential peripheral and deep margin assessment
CK: Cytokeratin
CT: Computed tomography
EMA: Epittielial membrane antigen
FDG: Fluorodeoxyglucose
Fig: Figure
Gy: Gray
Ki-67: Nuclcar- associated antigen Ki- 67
MMS: Mohs micrographic surgery
MRI: Magnetic resonance imaging
PAS: Periodicacid-schiff
SGC: Sweat gland carcinoma
